# Advances in approaches to study cell-type specific cortical circuits throughout development

**DOI:** 10.3389/fncel.2022.1031389

**Published:** 2022-10-17

**Authors:** Meretta A. Hanson, Jason C. Wester

**Affiliations:** Department of Neuroscience, The Ohio State University College of Medicine, Columbus, OH, United States

**Keywords:** neocortex, hippocampus, circuits, neuronal subtypes, transgenic mice, viral vectors, optogenetics

## Abstract

Neurons in the neocortex and hippocampus are diverse and form synaptic connections that depend on their type. Recent work has improved our understanding of neuronal cell-types and how to target them for experiments. This is crucial for investigating cortical circuit architecture, as the current catalog of established cell-type specific circuit motifs is small relative to the diversity of neuronal subtypes. Some of these motifs are found throughout the cortex, suggesting they are canonical circuits necessary for basic computations. However, the extent to which circuit organization is stereotyped across the brain or varies by cortical region remains unclear. Cortical circuits are also plastic, and their organization evolves throughout each developmental stage. Thus, experimental access to neuronal subtypes with temporal control is essential for studying cortical structure and function. In this mini review, we highlight several recent advances to target specific neuronal subtypes and study their synaptic connectivity and physiology throughout development. We emphasize approaches that combine multiple techniques, provide examples of successful applications, and describe potential future applications of novel tools.

## Introduction

Excitatory projection neurons and inhibitory interneurons are diverse and form local circuits that depend on their cell type. Currently, most studies of circuit organization identify neuronal types according to a taxonomy of major classes. Excitatory cells in the neocortex are parsed into three classes based on the target brain regions of their long-range axon: intratelencephalic (IT), pyramidal tract (PT), or corticothalamic (CT) (Harris and Shepherd, 2015). Excitatory cells in the hippocampus are parsed based on their depth within the stratum pyramidale as deep or superficial (Mizuseki et al., 2011; Slomianka et al., 2011; Marissal et al., 2012; Lee S. H. et al., 2014; Soltesz and Losonczy, 2018). Finally, inhibitory interneurons throughout neocortex and hippocampus are parsed into major classes based on expression of the molecular markers parvalbumin (PV), somatostatin (SST), or vasoactive intestinal peptide (VIP) (Tremblay et al., 2016; Pelkey et al., 2017). Several circuit motifs have been identified based on these broad neuronal classifications (Harris and Shepherd, 2015; Gutman-Wei and Brown, 2021). Among excitatory neurons, IT-type cells provide synaptic input to PT-type cells that is largely unreciprocated (Morishima and Kawaguchi, 2006; Brown and Hestrin, 2009; Kiritani et al., 2012). Among inhibitory interneurons, VIP+ cells preferentially inhibit SST+ cells (Pfeffer et al., 2013; Karnani et al., 2016). In deep layers of the neocortex, PV+ interneurons preferentially inhibit PT-type excitatory neurons (Lee A. T. et al., 2014; Ye et al., 2015; Wu et al., 2016), SST+ interneurons are preferentially targeted by PT-type cells (Le Be et al., 2007; Silberberg and Markram, 2007), and VIP+ interneurons are preferentially targeted by IT-type cells (Wester et al., 2019). Finally, in the CA1 region of hippocampus, PV+ interneurons preferentially inhibit deep excitatory pyramidal cells (Lee S. H. et al., 2014).

Such broad categorization of neuronal subtypes is necessary for investigating general features of circuit organization, synaptic physiology, and circuit function. However, each major neuronal class contains several distinct cell types that can be parsed according to additional morphological, electrophysiological, and molecular features (Kubota, 2014; Pelkey et al., 2017; Nigro et al., 2018; Huang and Paul, 2019; Kanari et al., 2019; Winnubst et al., 2019; Que et al., 2021). Recent single-cell RNA-sequencing studies provide further evidence for high diversity of both excitatory (Tasic et al., 2018; Chen et al., 2019; Kim et al., 2020; Cheung et al., 2021) and inhibitory (Tasic et al., 2018; Huang and Paul, 2019; Que et al., 2021) cell types defined by transcriptional profiles. Furthermore, within each major excitatory class, the subtypes defined by these profiles vary across the rostral to caudal poles of the neocortex (Saunders et al., 2018; Tasic et al., 2018; Bhattacherjee et al., 2019; Yao et al., 2021), and the ventral to dorsal poles of the hippocampus (Cembrowski et al., 2016; Cembrowski and Spruston, 2019). Thus, there may be a gradient of related yet distinct excitatory neuronal cell types across the neocortex and hippocampus (Cembrowski and Spruston, 2019; Yao et al., 2021). Such neuronal diversity raises important questions regarding circuit organization and development: To what extent does a circuit motif observed for a major neuronal class apply to each subtype represented within that class? Can circuit motifs involving major neuronal classes established in a specific brain region (e.g., visual cortex) be extrapolated to other, functionally distinct brain regions (e.g., prefrontal cortex)? How do circuit motifs change as cell types mature during development? Fortunately, several tools are now available to target and manipulate defined cell types to address such questions.

## Advances in genetic access to neuronal cell types will allow targeted approaches for investigating local circuits and their development

**FIGURE 1:**
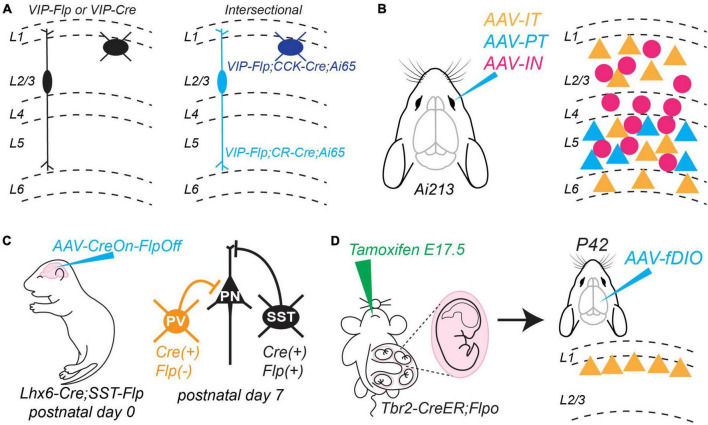
New methods to target and manipulate neuronal cell types. **(A)** Intersectional Cre-Flp approach to target neuronal subtypes within a major class. *Left*. Diverse subtypes of vasoactive intestinal peptide (VIP)-expressing interneurons are captured by the VIP-Flp and VIP-Cre mouse lines. *Right*. Subtypes can be parsed by their co-expression of VIP and cholecystokinin (CCK) or calretinin (CR). These can be targeted experimentally by crossing VIP-Flp mice with CCK-Cre or CR-Cre mice, respectively. The Ai65 mouse line expresses the fluorophore tdTomato dependent on expression of both Flp and Cre. Described in He et al. (2016). **(B)** New AAVs to deliver constructs driven by enhancers unique to different neuronal classes. Up to three different enhancer-driven AAVs can be delivered by retro-orbital injection to an Ai213 mouse, which encodes three conditional fluorophores controlled by unique recombinases. In this example, the AAVs drive expression in IT-type projection neurons, PT-type projection neurons, and inhibitory interneurons throughout cortex. Described in Graybuck et al. (2021). **(C)** Injecting an AAV encoding a construct that is expressed in the presence of Cre but suppressed in the presence of Flp allows intersectional targeting of interneuron subtypes that are otherwise inaccessible during early postnatal development. Here, the “parvalbumin (PV)” interneuron class does not yet express PV but can be targeted for experiments. Described in Pouchelon et al. (2021). **(D)** New tamoxifen-inducible Cre mouse lines generated by Matho et al. (2021) will allow different subtypes of excitatory projection neurons to be fate-mapped during embryogenesis and then studied during later postnatal development. In this example, a tamoxifen pulse at E17.5 to a pregnant Tbr2-CreER mouse causes transient translocation of Cre to the nucleus of cells that will eventually develop into layer 2 excitatory projection neurons. By further crossing this mouse to the Flpo line (He et al., 2016), transient Cre expression will be converted to permanent Flp expression (*left*). At later postnatal timepoints, an AAV encoding a Flp-dependent construct can be used to target and manipulate this neuronal subtype (*right*). Mouse cartoons in panels **(B–D)** modified from SciDraw (scidraw.io: doi: 10.5281/zenodo.3925903; doi: 10.5281/zenodo.3926569; doi: 10.5281/zenodo.3926600).

The generation of transgenic mice and AAVs that express Cre or Flp recombinase under the control of promoters or enhancers specific to unique neuronal subtypes is a major advance for studying microcircuits. Several currently available mouse lines target Cre or Flp expression to different populations of excitatory projection neurons (Gerfen et al., 2013; Daigle et al., 2018; Matho et al., 2021; Vaasjo et al., 2022) and inhibitory interneurons (Taniguchi et al., 2011; He et al., 2016). Furthermore, several labs are expanding available AAVs for cell-type specific targeting (Nair et al., 2020; Vormstein-Schneider et al., 2020; Graybuck et al., 2021; Mich et al., 2021; Pouchelon et al., 2022). Experimental strategies that combine Cre and Flp refine targeting of cell types in an intersectional manner by considering multiple genetic features (Fenno et al., 2014, 2017). For example, VIP+ interneurons can be parsed into two subtypes with unique morphological and electrophysiological profiles based on co-expression of calretinin (CR) or cholecystokinin (CCK) (Cauli et al., 1997, 2014; Kawaguchi and Kubota, 1998; He et al., 2016). He et al. (2016) successfully targeted these distinct VIP/CR and VIP/CCK subtypes by crossing VIP-Flp mice to CR-ires-Cre or CCK-ires-Cre mice and an Ai65 dual conditional Cre/Flp reporter line (Figure 1A). Advances in designing AAV vectors promise to make such intersectional experiments more tractable (Nair et al., 2020; Vormstein-Schneider et al., 2020; Graybuck et al., 2021; Mich et al., 2021; Pouchelon et al., 2022). For example, Graybuck et al. (2021) generated AAVs that use cell-type specific enhancers to drive expression of Cre, Flp, or Nigri recombinases. They optimized these AAVs for retro-orbital injection, which results in wide-spread expression in the brain without the need for invasive, direct stereotaxic injection. Finally, they generated a new transgenic mouse line, Ai213, which is a triple conditional reporter with different fluorophores independently controlled by Cre, Flp, and Nigri expression. Using these tools, they successfully tagged PT-type cells, IT-type cells, and interneurons with unique fluorophores throughout the cortex without the need for complicated transgenic crosses (Figure 1B). Finally, Pouchelon et al. (2022) developed a set of AAV backbones that allow for boolean intersectional experiments that include: Cre-ON, Flp-ON, Cre-ON;Flp-ON, Cre-ON;Flp-OFF, and Cre-OFF;Flp-ON. These backbones can be integrated with several existing technologies (e.g., optogenetics, calcium-indicators, and DREADDs) and then used with available conditional transgenic mice. These technologies will greatly simplify experiments to study synaptic connectivity among multiple neuronal classes and will eventually allow intersectional approaches to target specific neuronal subtypes within a major class.

These methods can also be applied to study how distinct neuronal subtypes mature and circuit motifs form during development. This can be challenging because molecular genetic markers used to define neuronal classes may not be expressed during early developmental stages. For example, PV expression begins after the second postnatal week, which limits the utility of the PV-Cre mouse line for targeting this interneuron class during synapse formation. However, a Cre-ON/Flp-OFF strategy can circumvent this limitation (Pouchelon et al., 2021). PV+ and SST+ interneurons are generated from a common progenitor pool that expresses the transcription factor Lhx6 beginning during embryogenesis (Flandin et al., 2011; Pelkey et al., 2017). Thus, to target interneurons at postnatal day 10 that do not yet express PV, Pouchelon et al. (2021) crossed Lhx6-Cre mice to SST-Flp mice and injected an AAV encoding a Flp-OFF construct at birth, thereby limiting labeling to Lhx6+ cells without SST (Figure 1C). This allowed them to perform monosynaptic rabies tracing (discussed in detail below) to map early presynaptic inputs to these cells. Alternatively, two groups recently developed AAVs that take advantage of enhancer elements to target this interneuron class prior to the expression of PV (Vormstein-Schneider et al., 2020; Mich et al., 2021). Importantly, Vormstein-Schneider et al. (2020) found that local cortical injection of AAV-E2-tdTomato allowed them to identify fast-spiking (future PV-expressing) interneurons as early as postnatal day 7. Thus, a simple viral vector strategy is now available to target the PV+ class of interneurons during early development to understand how they integrate into circuits.

Some neuronal subtypes can be defined by temporally limited expression of key transcription factors during embryogenesis. For example, using a tamoxifen-inducible Nkx2.1-CreER mouse line (Taniguchi et al., 2011), Taniguchi et al. (2013) found that neocortical axo-axonic chandelier cells can be targeted based on their late birthdate during embryogenesis. Until recently, transgenic strategies to target excitatory projection neurons in the neocortex and hippocampus were largely limited to use of the Ngn2-CreER mouse line (Zirlinger et al., 2002; Marissal et al., 2012). Projection neurons express Neurogenin2 as they become postmitotic; thus, tamoxifen pulses timed to early or late embryogenesis capture classes unique to different neocortical and hippocampal lamina. However, early born neocortical neurons are a mix of IT, CT, and PT classes, which limits the utility of this strategy for studying their independent development. To overcome this limitation, Matho et al. (2021) recently generated several tamoxifen-inducible Cre mice that allow embryonic temporal fate-mapping of excitatory subtypes within each major class. An exciting future approach is to cross these mice to LSL-Flpo mice (He et al., 2016), which converts transient Cre expression into permanent Flp expression (Cre to Flp conversion). Thus, fate-mapped excitatory neurons can be manipulated at later developmental times by injecting AAVs encoding Flp-dependent constructs (Figure 1D). These tools will be crucial for investigating how different excitatory neuronal subtypes choose their synaptic partners during development.

## Novel strategies that apply monosynaptic circuit mapping will reveal candidate circuit motifs underlying local computations

**FIGURE 2:**
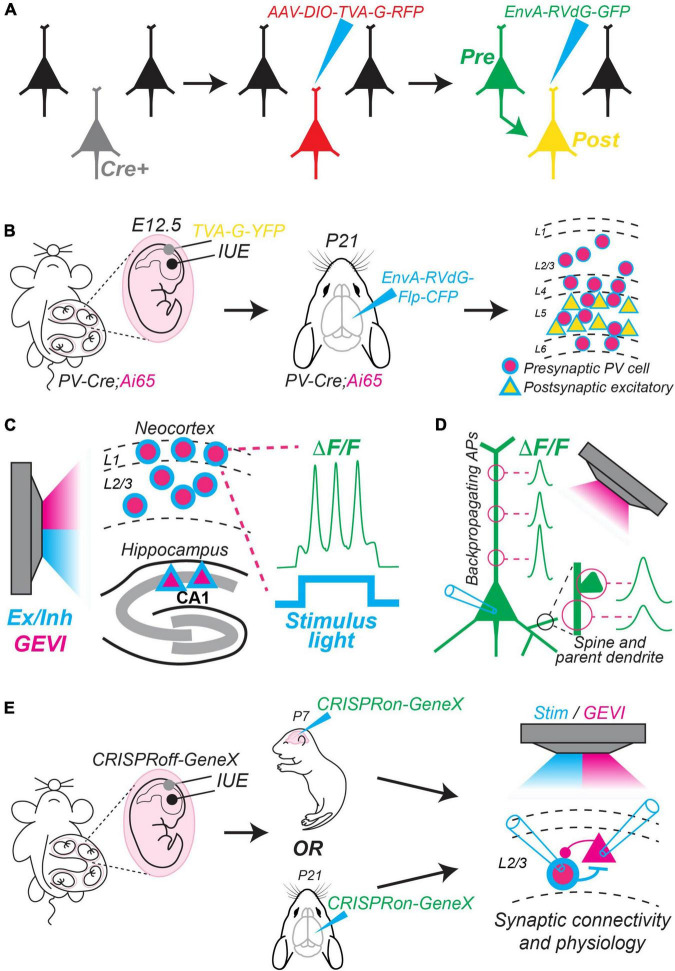
New methods to map and manipulate circuit motifs. **(A)** Conventional method of monosynaptic retrograde rabies tracing. First, an appropriate Cre driver mouse line is chosen. Second, an AAV (helper virus) is injected that encodes a Cre-dependent construct containing TVA, G, and red fluorescent protein (RFP). Finally, pseudotyped g-deleted rabies (EnvA-RVdG) encoding green fluorescent protein (GFP) is injected. Only Cre-expressing cells previously infected with the AAV express TVA, which allows them to be further infected by EnvA-RVdG. Thus, they express red and green fluorophores (yellow). The yellow cells also express the missing G, which allows EnvA-RVdG to travel retrograde to label presynaptic cells in green. Green cells lack G, so spread of the rabies virus stops. **(B)** Summary of experimental steps used by Yetman et al. (2019) to map connections between excitatory and inhibitory neurons using a Cre/Flp intersectional approach combined with monosynaptic rabies tracing. In this example, PV+ interneurons that are presynaptic to deep layer excitatory pyramidal cells are mapped. They used a PV-Cre mouse crossed to an Ai65 dual Cre/Flp conditional reporter line encoding RFP. First, they performed *in utero* electroporation (IUE) at embryonic day 12.5 (E12.5) to deliver a plasmid encoding TVA, G, and yellow fluorescent protein (YFP) to progenitors of deep layer neocortical projection neurons. Second, they injected EnvA-RVdG encoding Flp and cyan fluorescent protein (CFP) at postnatal day 21 (P21). This results in deep layer excitatory starter neurons that express both cyan fluorescent protein (CFP) and yellow fluorescent protein (YFP); presynaptic PV+ interneurons express both RFP and CFP. **(C)** Different neuronal subtypes (e.g., neocortical interneurons or hippocampal CA1 pyramidal cells) can be made to simultaneously express a genetically encoded voltage indicator (GEVI) (pink) and a blue-shifted optogenetic activator or inhibitor for concurrent stimulation and imaging. In this example, blue light stimulates channelrhodopsin to elicit action potentials, which are imaged from the soma of a L1 interneuron [see Fan et al. (2020)]. **(D)** GEVIs allow imaging of dendritic compartments and spines with high temporal resolution. For example, Chien et al. (2021) imaged backpropagating action potentials at different locations along the apical dendrite (left). Cornejo et al. (2022) found differences in voltage responses measured simultaneously in spines and their parent dendrite (bottom right). **(E)** Potential strategy to use CRISPRoff/on (Nuñez et al., 2021) to study the function of genes that begin expression during early development and continue to be expressed throughout postnatal periods. First, CRISPRoff could be delivered *via in utero* electroporation (IUE) to silence a gene of interest (GeneX) during early embryogenesis. Second, CRISPRon could be delivered *via* a viral vector injected at different postnatal timepoints to reinstate activity of GeneX. Finally, circuit motifs could be studied in the presence or absence of GeneX at these different timepoints using monosynaptic rabies tracing, optogenetic stimulation, optical imaging using GEVIs, and/or traditional synaptic physiology. Mouse cartoons in panels **(B,E)** modified from SciDraw (scidraw.io: doi: 10.5281/zenodo.3925903; doi: 10.5281/zenodo.3926569; doi: 10.5281/zenodo.3926600).

A powerful approach to study microcircuit motifs is to combine tools that offer improved genetic access to different neuronal subtypes with monosynaptic rabies tracing (Wickersham et al., 2007). In brief, this technique uses a modified rabies virus (RV*d*G) missing a key glycoprotein (G) necessary for retrograde transmission after initial infection. This virus (RV*d*G) is pseudotyped with an avian envelop protein EnvA, thus, it can only infect cells that express the avian receptor TVA [reviewed in detail in Callaway and Luo (2015)] (Figure 2A). Recent advances in this technique incorporate Cre/Flp intersectional approaches and new sfluorophores for combinatorial labeling. For example, Yetman et al. (2019) used this strategy to map inputs to different populations of excitatory projection neurons from major classes of inhibitory interneurons (Figure 2B). To target starter excitatory neurons in different cortical layers, they performed *in utero* electroporation (IUE) to deliver a plasmid encoding TVA, G, and a yellow fluorescent protein (YFP) at embryonic timepoints unique to the generation of superficial or deep layer cells. To target major interneuron classes, they used PV-Cre, SST-Cre, and VIP-Cre mice crossed to an Ai65 mouse, which is a dual Cre/Flp conditional red fluorescent protein (RFP) reporter. Thus, their strategy was to use IUE to transfect embryos of PV-Cre:Ai65, SST-Cre:Ai65, or VIP-Cre:Ai65 mice with TVA + G + YFP plasmids on embryonic days 12.5 or 15.5 to target superficial or deep layer excitatory starter neurons, respectively. Finally, during the third postnatal week, they injected EnvA-RV*d*G encoding Flp and cyan fluorescent protein (CFP). As a result, they labeled starter excitatory neurons within layers 2–3 or 4–6 with YFP/CFP and presynaptic interneurons (PV+, SST+, or VIP+) with RFP/CFP. Thus, they established a high throughput screen to discover local microcircuits among major excitatory and inhibitory neuronal classes. This study provides a template for using creative intersectional approaches in combination with monosynaptic rabies tracing to reveal new circuit motifs.

There are important limitations and caveats to consider when using RV*d*G to trace circuits. These include non-selective neuronal labeling (false positives) and lack of viral transmission at some synapses (false negatives) [for review see Rogers and Beier (2021)]. Infection with RV*d*G also leads to changes in expression of several genes, including those related to synaptic transmission (Huang and Sabatini, 2020; Patiño et al., 2022). However, even if this technique reveals only a subset of circuit motifs, this information is invaluable to guide targeted experiments. Furthermore, combining rabies tracing with single-cell RNA-sequencing has the potential to specify sets of unique neuronal subtypes within major classes that contribute to local microcircuit motifs. Indeed, Patiño et al. (2022) found that despite RV*d*G-induced gene expression changes, it is possible to identify neuronal subtypes according to transcriptomic profiles previously identified by the Allen Institute for Brain Science (Tasic et al., 2018). Thus, presynaptic cells labeled by RV*d*G can be sorted by fluorescence, followed by single-cell or single-nucleus RNA-sequencing to map neurons to unique subtypes. Once candidate circuits are identified, future studies can use electrophysiological and optogenetic techniques to investigate subtype-specific synaptic connectivity and synaptic physiology.

## Cell-type specific expression of genetically encoded voltage indicators will allow high throughput study of circuits *in vitro* and *in vivo*

Visualized dual whole-cell patch clamp recording in brain slices remains the gold standard for studying detailed synaptic physiology of unitary connections between identified neurons in local circuits (Campagnola et al., 2022). However, this technique requires that connected neurons be within proximity of 100–200 microns, traditionally measures synaptic currents at the soma, and is impractical *in vivo*. Recent advances in genetically encoded voltage indicators (GEVIs) provide exciting new opportunities to perform “optical electrophysiology.” GEVIs offer excellent temporal resolution compared to calcium indicators, report action potentials and subthreshold responses *in vivo*, and can be combined with optogenetic stimulation or inhibition (Adam et al., 2019; Piatkevich et al., 2019; Fan et al., 2020; Bando et al., 2021; Cornejo et al., 2022). Furthermore, GEVIs can be expressed in specific neuronal subtypes *via in utero* electroporation, injection of AAVs encoding floxed constructs, or by using the ArcLight mouse line, which expresses a Cre/Tet-dependent indicator (Daigle et al., 2018; Platisa et al., 2022). With these advances, it is now possible to both control and record neuronal membrane potential from identified cell-types in awake mice during behavioral and perceptual tasks. For example, Fan et al. (2020) imaged inhibitory interneurons in layer 1 of neocortex while optogenetically controlling membrane voltage to assay excitatory and inhibitory input to these cells during whisker stimulation (Figure 2C). GEVIs also allow measurement of voltage responses in membrane compartments that are difficult or impossible to access for whole-cell patch clamp recording. Chien et al. (2021) imaged backpropagating action potentials at multiple locations along the apical dendrite simultaneously, and Cornejo et al. (2022) simultaneously imaged spines and their parent dendrites in mice *in vivo* to determine if they exhibit independent voltage responses (Figure 2D). Thus, combined imaging and optogenetic techniques hold tremendous promise for the study of cortical circuits and synaptic physiology.

Genetically encoded voltage indicator technology is advancing rapidly, and there are many variants with advantages and disadvantages unique to their design (Bando et al., 2019b; Milosevic et al., 2020). There are two broad categories of GEVIs: voltage-sensing domain (VSD)-based and rhodopsin-based [reviewed in detail in Adam (2021), Bando et al. (2019a), Knöpfel and Song (2019)]. Rhodopsin-based GEVIs offer high signal-to-noise ratio and excellent kinetics and have been successfully used *in vitro* to image dendritic compartments and *in vivo* to image surface-level cells using 1-photon imaging (Fan et al., 2020; Chien et al., 2021) (see Figures 2C,D). However, they are dim and not currently suitable for deep tissue imaging using 2-photon microscopy. VSD-based GEVIs offer comparatively lower signal-to-noise ratio and slower kinetics but are currently the best option for measuring responses *in vivo*. Among these, the ASAP family is very promising (Villette et al., 2019), and the most recent variant, JEDI-2P, offers state-of-the-art kinetics, signal-to-noise ratio, and photostability under 2-photon excitation (Liu et al., 2022). For example, JEDI-2P allowed successful *in vivo* 2-photon imaging of action potentials from neurons in neocortical layer 5 and long-duration dual recording of neighboring neurons in layer 2/3 (Liu et al., 2022). As both VSD- and rhodopsin-based GEVIs improve, they will allow advanced optical interrogation of membrane voltage of specific neuronal subtypes *in vivo*.

## New epigenetic editing techniques will allow the mechanisms of circuit formation to be investigated and manipulated with greater precision and temporal control

As new circuit motifs are discovered, an important next step will be to understand the molecular mechanisms underlying their development and maintenance. Novel approaches using CRISPR with catalytically inactivated Cas enzymes (dCas9) may offer powerful experimental tools for this challenge. When fused with the transcriptional repressor KRAB or the activator VP64, dCas9 induces epigenetic alterations to gene expression (Qi et al., 2013; Holtzman and Gersbach, 2018; Nakamura et al., 2021; Hu and Li, 2022). Thus, CRISPR interference (CRISPRi) can be used to silence gene expression and CRISPR activation (CRISPRa) to enhance gene expression (Gilbert et al., 2013; Maeder et al., 2013; Perez-Pinera et al., 2013; Yim et al., 2020). For example, Zheng et al. (2018) used CRISPRi to conditionally knockdown *syt1*, which encodes synaptotagmin 1, a protein necessary for neurotransmitter release. They created novel lentiviruses encoding a single guide RNA for *syt1* and dCas9-KRAB under the control of the promoters CaMKIIa or VGAT to target glutamatergic or GABAergic neurons, respectively. Injecting these vectors into the hippocampal dentate gyrus of mice significantly reduced evoked glutamatergic or GABAergic synaptic currents in neighboring neurons and differentially affected memory acquisition. With the discovery of new cell-type specific enhancers and promotors, CRISPRi and CRISPRa may be applied to study gene function in distinct neuronal types, in specific brain regions, and at multiple developmental time points.

CRISPR interference and CRISPRa are powerful tools, but they do face some limitations. Constitutive expression is required to maintain gene silencing or activation and cannot be reversed. Recently, Nuñez et al. (2021) introduced a new form of CRISPR-based epigenetic editing, termed CRISPRoff, and CRISPRon to address these issues. CRISPRoff requires only transient expression to induce stable knockdown of a target gene *via* DNA methylation. Furthermore, CRISPRoff is multiplexable, allowing reliable silencing of up to three genes within a single cell. It is also heritable, and thus can be targeted to neural progenitors. Importantly, epigenetic silencing can be reversed by CRISPRon, which removes DNA methylation and recruits transcriptional machinery to loci impacted by CRISPRoff. While this technology has only been tested in cultured cells, it may eventually offer temporally resolved and reversible control of gene expression within specific neuronal subtypes. For example, it could be used to investigate transcription factors that control neuronal differentiation during early development but then remain active into adulthood. An exciting possibility is to use CRISPRoff to silence these genes during embryogenesis, followed by delivery of CRISPRon to reinstate expression at multiple later developmental stages to study how their function evolves over time. Such experiments could temporally resolve cell fate decisions that impact synaptic connectivity and circuit motif assembly (Figure 2E).

A hurdle for employing any CRISPR-based technology is safe and efficient delivery to cells of interest *in vivo* (Wang et al., 2020; Yip, 2020; Horodecka and Duchler, 2021). Vectors must encode large constructs that contain a cell-type specific promoter, Cas9, and the necessary single guide RNA(s) for target genes. This is particularly challenging for designing experiments that combine CRISPRoff and CRISPRon to rescue transient gene knockdown because each construct is approximately 7 kb (Nuñez et al., 2021). AAVs are currently the most popular vectors for *in vivo* gene delivery given their safety profile; however, they have a limited capacity of 4.7 kb (Grieger and Samulski, 2005; Wu et al., 2010; Wang et al., 2020). To utilize AAVs, a potential solution is to split the constructs among multiple vectors to be injected at a single site (Hirsch et al., 2010; Truong et al., 2015; Wang et al., 2020). However, each AAV vector would need to be taken up by the same cell, which may limit the efficacy of this approach (Wang et al., 2020). Lentivirus is currently the best vector option, due to its ∼8–10 kb capacity, but multiple vectors may still be required depending on the experimental construct (Sanjana et al., 2014; Shalem et al., 2014; Zheng et al., 2018; Savell et al., 2019). Current lentiviral vectors integrate into the host genome, which can be advantageous for studying development but raises concerns regarding insertional mutagenesis. However, non-integrating vectors are in development (Luis, 2020), thus lentivirus may remain the best *in vivo* delivery strategy for CRISPRoff and CRISPRon. An alternative to viral vectors is to use *in utero* electroporation (IUE) to deliver a plasmid encoding a CRISPR-based construct to specific neuronal types during embryonic development. Indeed, IUE has been used to successfully deliver such constructs to excitatory neurons in layer 2/3 of the cerebral cortex (Shinmyo et al., 2016) and apical radial glial cells in the developing neocortex (Kalebic et al., 2016). Thus, IUE could deliver CRISPRoff to specific neuronal types during embryonic development, but a viral vector would be required to subsequently deliver CRISPRon during later postnatal periods. In summary, a successful delivery approach must be established before CRISPRoff and CRISPRon can be fully utilized *in vivo*.

## Concluding remarks

This is an exciting time to study cortical microcircuits and their development. Several new tools can be used in combination to target neurons with cell type specificity, map their synaptic connections, and study the molecular mechanisms underlying how they choose their synaptic partners. Furthermore, these tools can be applied across developmental stages to study cortical circuit assembly and plasticity. Optimistically, it appears the field is on the verge of defining a “parts list” of cell-type specific microcircuit motifs that will help us to better understand how the cortex functions under both healthy and pathological conditions.

## Author contributions

MH and JW wrote and edited the manuscript. Both authors contributed to the article and approved the submitted version.
